# Room-Temperature Synthesis of Carbon-Nanotube-Interconnected Amorphous NiFe-Layered Double Hydroxides for Boosting Oxygen Evolution Reaction

**DOI:** 10.3390/molecules28217289

**Published:** 2023-10-27

**Authors:** Zhuo Chen, Qiang Qu, Xinsheng Li, Katam Srinivas, Yuanfu Chen, Mingqiang Zhu

**Affiliations:** 1 College of Mechanical and Electronic Engineering, Northwest A&F University, Yangling 712100, China; 2 State Key Laboratory of Electronic Thin Films and Integrated Devices, School of Integrated Circuit Science and Engineering, University of Electronic Science and Technology of China, Chengdu 610054, China

**Keywords:** room-temperature synthesis, layered double hydroxides, carbon nanotubes, NiFe-LDH@CNT, oxygen evolution reaction

## Abstract

The oxygen evolution reaction (OER) is a key half-reaction in electrocatalytic water splitting. Large-scale water electrolysis is hampered by commercial noble-metal-based OER electrocatalysts owing to their high cost. To address these issues, we present a facile, one-pot, room-temperature co-precipitation approach to quickly synthesize carbon-nanotube-interconnected amorphous NiFe-layered double hydroxides (NiFe-LDH@CNT) as cost-effective, efficient, and stable OER electrocatalysts. The hybrid catalyst NiFe-LDH@CNT delivered outstanding OER activity with a low onset overpotential of 255 mV and a small Tafel slope of 51.36 mV dec^−1^, as well as outstanding long-term stability. The high catalytic capability of NiFe-LDH@CNT is associated with the synergistic effects of its room-temperature synthesized amorphous structure, bi-metallic modulation, and conductive CNT skeleton. The room-temperature synthesis can not only offer economic feasibility, but can also allow amorphous NiFe-LDH to be obtained without crystalline boundaries, facilitating long-term stability during the OER process. The bi-metallic nature of NiFe-LDH guarantees a modified electronic structure, providing additional catalytic sites. Simultaneously, the highly conductive CNT network fosters a nanoporous structure, facilitating electron transfer and O_2_ release and enriching catalytic sites. This study introduces an innovative approach to purposefully design nanoarchitecture and easily synthesize amorphous transition-metal-based OER catalysts, ensuring their cost effectiveness, production efficiency, and long-term stability.

## 1. Introduction

The extensive consumption of traditional energy resources and their environmental impact have propelled the significant exploration of renewable energy technologies in recent years [1,2,3,4]. One particular area of interest is electrochemical water splitting, emerging as a leading approach for acquiring clean hydrogen energy [5]. In this process, the oxygen evolution reaction (OER) holds paramount significance [6]. However, the sluggish kinetics of the OER have impeded the widespread adoption of large-scale water electrolysis [7,8]. Therefore, it is urgent to develop highly efficient and stable catalysts to mitigate the reaction barrier of the OER.

Although commercial noble-metal-based catalysts exhibit good OER performance, the widespread utilization of these catalysts is mainly constrained by their high cost, scarcity, and slow kinetics [9,10,11]. Recently, non-noble transition-metal-based compounds have shown significant promise as electrocatalysts for the OER [12]. Among them, NiFe-based layered double hydroxides (LDHs) have gained great attention, especially due to the disparity in the electron affinity and ionic radius between Ni and Fe, which could create a local potential difference and facilitate reaction kinetics [13]. However, the synthesis method and catalytic activity of LDH catalysts should be significantly improved.

Generally, LDHs can be synthesized via various methods, such as hydrothermal reactions [14], solvothermal reactions [15], or co-precipitation methods [16], at elevated temperatures for a long time. Such syntheses involving elevated temperatures and extended durations are energy-intensive and operationally complex, thus increasing production costs. Therefore, it is necessary to develop a facile and rapid synthesis approach at low temperatures to solve the issues of high energy consumption, a complicated operation, and high production costs. In addition, the control of amorphous LDH structures during synthesis is very important because an amorphous catalyst without crystalline boundaries generally has better long-term stability [17]. Electronic modulation and conductive skeleton incorporation have been considered as efficient strategies for catalytic performance enhancement. The bi-metallization of LDHs delivers much better OER performance than monometallic LDHs due to the modulation of the electronic structure and the presence of richer active sites [18]. Several groups have reported that introducing conductive carbon nanomaterials can effectively mitigate the low conductivity issue of LDHs, thus improving the OER performance [19,20]. However, it is still challenging to combine a facile synthesis method and synergistic OER performance enhancement for LDHs with a low production cost, a controllable crystalline nature, high efficiency, and long-term stability. 

To address such issues, herein, we present a facile, one-pot, room-temperature co-precipitation method to synthesize CNT-interconnected amorphous NiFe-LDH (NiFe-LDH@CNT) as an efficient and stable OER catalyst. This room-temperature synthesis technology has several advantages, such as rapid synthesis, economic feasibility, environmental sustainability, and easy control of the amorphous nature, compared to conventional LDH synthesis methods at elevated temperatures for a long time. Furthermore, the amorphous nature of NiFe-LDH facilitates long-term stability during the OER process; the bi-metallic nature of NiFe-LDH guarantees a modified electronic structure with additional active sites; and the highly conductive CNT network promotes a nanoporous structure with an enhanced surface area and rich catalytic sites, and provides efficient conductive paths and a well-established interface between the CNTs and the LDHs. Due to the synergistic effects mentioned above, the amorphous NiFe-LDH@CNT catalyst delivered an outstanding OER performance, showcasing a low onset overpotential (255 mV) and Tafel slope (51.36 mV dec^−1^) as well as significant durability. This study outlines an approach for the deliberate nanoarchitecture design and facile synthesis of amorphous LDH catalysts for the OER, focusing on cost effectiveness, production efficiency, and long-term stability.

## 2. Results and Discussion

### 2.1. Synthesis and Structural Characterizations

**Figure 1:**
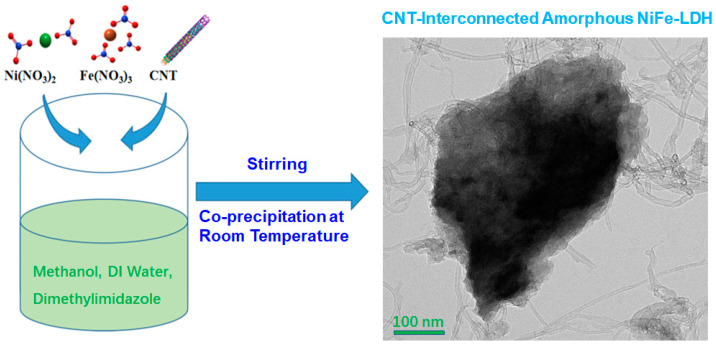
Schematic illustration of NiFe-LDH@CNT fabrication.

As schematically illustrated in Figure 1, the Ni-LDH, Fe-LDH, NiFe-LDH, and NiFe-LDH@CNT samples were prepared using a facile, one-pot, co-precipitation approach at room temperature. A CNT slurry was used to build a functional network with excellent conductivity, a stable structure, and high reaction activity. 

**Figure 2:**
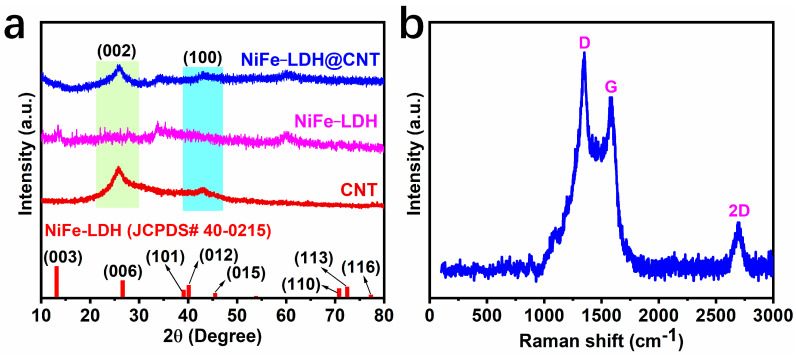
(**a**) XRD patterns of NiFe-LDH@CNT, NiFe-LDH, and commercial CNTs. (**b**) Raman spectrum of NiFe-LDH@CNT.

In order to investigate the crystalline structures of the samples, the X-ray diffraction (XRD) patterns of commercial carbon nanotubes (CNTs), NiFe-LDH, and NiFe-LDH@CNT were recorded. As depicted in Figure 2a, the CNT sample showed a clear (002) peak around 26° and a few weak peaks appeared on the pattern of NiFe-LDH, indicating its lack of discernible crystalline structures; as for NiFe-LDH@CNT, a characteristic peak around 26° was indexed as the (002) peak of CNTs, and there were nearly no obvious peaks for NiFe-LDH, indicating its amorphous nature or lack of discernible crystalline structures, which was mainly attributed to the low-temperature synthesis at room temperature. The amorphous nature of NiFe-LDH in NiFe-LDH@CNT means that there was a lack of crystalline boundaries, which suggests that NiFe-LDH@CNT might be more stable in alkaline electrolytes during the OER, comparable to a crystalline sample. The Raman spectrum of NiFe-LDH@CNT was characterized. Figure 2b illustrates three distinct peaks at 1350.3, 1574.0, and 2690.5 cm^−1^, which represent the D, G, and 2D bands of the CNTs, respectively. A large I_D_/I_G_ ratio of 1.26 and a distinct 2D peak in the Raman spectrum indicated the presence of significant lattice defects and a higher degree of graphitization after 5% CNT incorporation. This suggests that NiFe-LDH@CNT could have abundant active sites and high conductivity.

**Figure 3:**
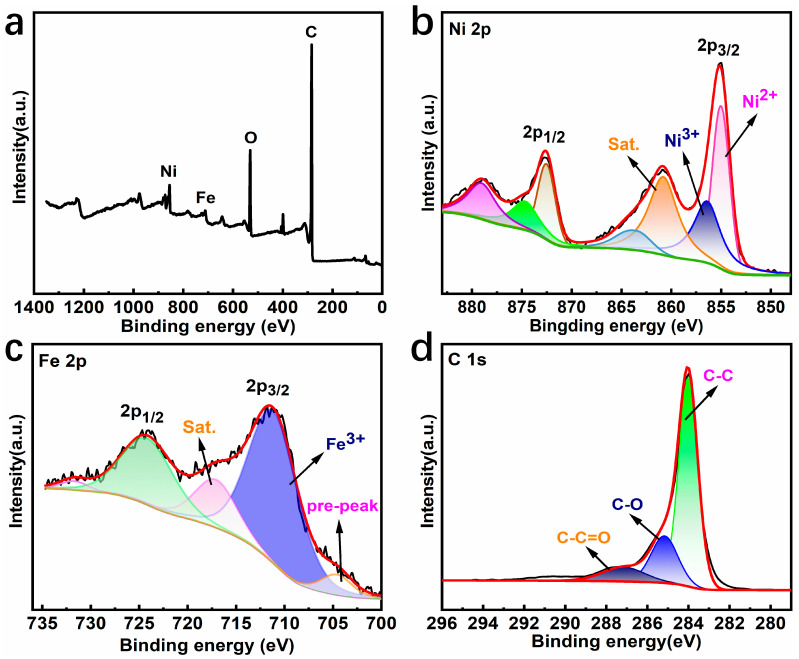
XPS spectral data of NiFe-LDH@CNT: (**a**) survey spectrum and high-resolution spectra of (**b**) Ni 2p, (**c**) Fe 2p, and (**d**) C 1s.

X-ray photoelectron spectroscopy (XPS) was employed to gain insights into the surface microstructure and chemical composition of NiFe-LDH@CNT. As shown in Figure 3a, the characteristic peaks of the Ni, Fe, O, and C elements appeared in its survey spectrum, which confirms the co-existence of these elements. Further detailed insights into the Ni 2p, Fe 2p, and C 1s spectra of NiFe-LDH@CNT are illustrated in Figure 3b–d, respectively, with the corresponding peak-fitting data summarized in Table S1. 

The XPS spectra of the Ni and Fe elements can be split into two doublet components (2p_3/2_ and 2p_1/2_) as a result of spin-orbit coupling. Figure 3b,c illustrates that, for NiFe-LDH@CNT, Ni ions predominantly resided in the oxidation state of Ni^2+^, while Fe ions primarily occupied the +3 oxidation state. The peaks located at 854.99 and 872.52 eV denote the Ni^2+^ state, while the additional peaks at 856.40 and 874.68 eV denote the Ni^3+^ state, and these are accompanied by three satellite peaks located at around 860.80, 863.46, and 879.03 eV. Similarly, during the fitting analysis of the Fe 2p peak shown in Figure 3c, a characteristic pair of peaks at the binding energies of 711.46 and 724.63 eV were detected. These peaks were assigned to Fe^3+^ states with electron configurations of 2p_3/2_ and 2p_1/2_ along with a pair of shake-up satellite peaks. In addition, a distinct low-intensity peak located at 704.67 eV was observed preceding the 2p_3/2_ peak, indicating the presence of lower-oxidation-state Fe ions in the vicinity (Figure 3c). This is very consistent with previous research [21,22]. The valence states on the material’s surface were found to be unaffected by the presence of the CNT networks. This finding was further supported by the high-resolution spectrum of C1s (Figure 3d). 

**Figure 4:**
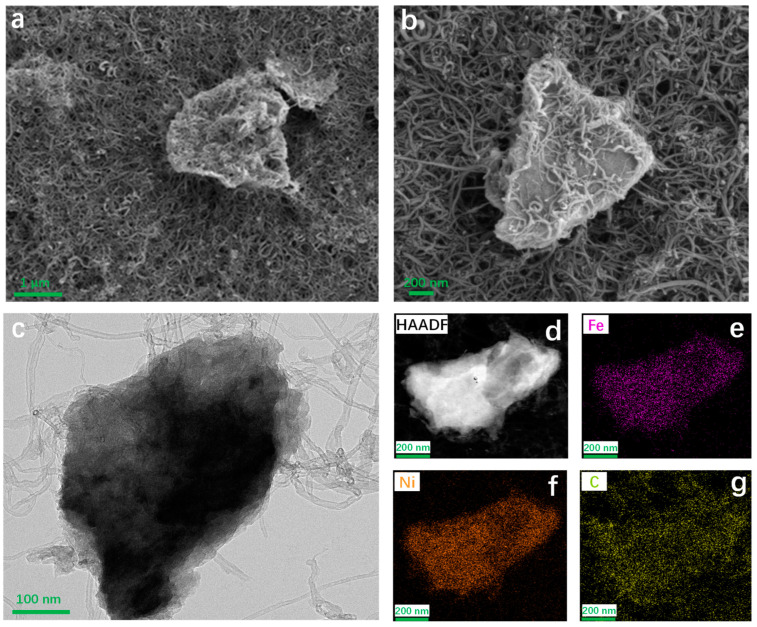
(**a**) SEM, (**b**) TEM, (**c**) HR-TEM, and (**d**–**g**) elemental mapping images of NiFe-LDH@CNT.

Further characterization of the surface morphology and structural features of NiFe-LDH@CNT was carried out using scanning electron microscopy (SEM) and transmission electron microscopy (TEM). Figure 4a,b presents the SEM and TEM images, highlighting the presence of carbon nanotubes and NiFe-LDH particles. From the high-resolution TEM (HR-TEM) image of NiFe-LDH@CNT (Figure 4c), one can clearly observe that the NiFe-LDH nanoparticle was interconnected by many carbon nanotubes. This interconnection suggests the successful integration of conductive carbon nanotubes, which is anticipated to effectively facilitate electron transfer in the NiFe-LDH@CNT composite. Figure 4d–g shows the morphology and corresponding elemental mapping images. The consistency between the morphology and element mapping indicates a high level of uniformity and ordered atomic arrangement at the nanoscale level. 

### 2.2. Oxygen Evolution Activity 

**Figure 5:**
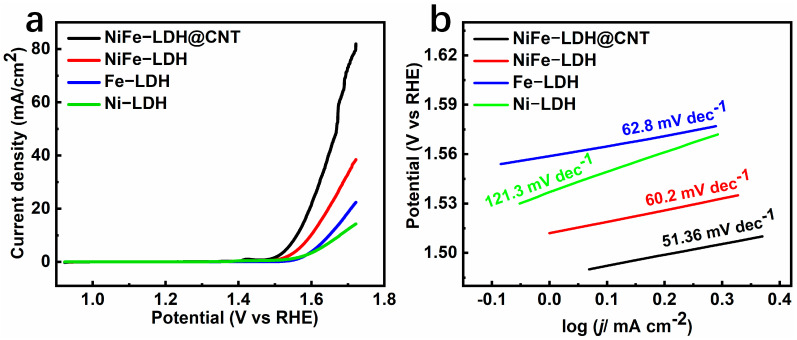
(**a**) LSV plots of NiFe-LDH@CNT, NiFe-LDH, Fe-LDH, and Ni-LDH, and (**b**) the corresponding Tafel slopes.

To assess the OER performance of the as-synthesized catalysts, a standard three-electrode setup with a 1.0 M KOH electrolyte was utilized. Initially, an activated glassy carbon electrode (GCE) was employed for linear sweep voltammetry (LSV) testing, which offered insights into the catalytic activity, reaction mechanisms, and electrocatalytic performance of the materials [23]. During the electrochemical reactions, chemical reactions occurred on the surface of the material when an external potential was applied [24]. As shown in Figure 5a, when a fixed potential (particularly larger than 1.6 V) was applied, NiFe-LDH obtained a larger current density than that of Ni-LDH or Fe-LDH, which suggests that bi-metallization can improve the catalytic activity. Furthermore, the incorporation of conductive CNTs in NiFe-LDH@CNT resulted in a much higher current density than that of NiFe-LDH, confirming that, after introducing conductive carbon nanotubes, the catalytic activity was significantly enhanced. The onset overpotentials at 1 mA cm^−2^ for Ni-LDH, Fe-LDH, NiFe-LDH, and NiFe-LDH@CNT were 329, 307, 282, and 255 mV, respectively. Obviously, compared to the monometallic LDHs of Ni-LDH (329 mV) and Fe-LDH (307 mV), bi-metallic NiFe-LDH had a much lower onset overpotential (282 mV), suggesting that bi-metallization is beneficial for decreasing the overpotential due to modifications of the electronic structure. Furthermore, one can observe that, compared to NiFe-LDH, the onset overpotential of NiFe-LDH@CNT can be significantly decreased. Similarly, to achieve the benchmark 10 mA cm^−2^ current density, NiFe-LDH@CNT required a much lower overpotential (*η*_10_ = 330 mV) compared to the other samples (448 mV for Ni-LDH, 419 mV for Fe-LDH, and 369 mV for NiFe-LDH). This indicates that the excellent OER activity could be owing to the synergistic effect of bi-metallization and the introduction of a conductive CNT network.

To gain a deeper understanding, Tafel slope calculations were conducted using the LSV data. The Tafel slope offers insights into reaction feasibility, which can be calculated from either the Tafel equation (*η* = a + b × log(*j*)) or *E* = a_1_ + b × log(*j*). According to the widely acknowledged mechanism, the electrocatalytic efficiency of the OER is closely associated with the energy barriers encountered during the conversion of OH* to O* and O* to OOH* [25]. The Tafel slope, as determined by applying the Butler–Volmer theory, serves as an indicator for identifying the rate-determining step in each reaction stage [26]. The Tafel slope can be used to further assess the OER kinetics. As shown in Figure 5b, it is evident that, compared to the monometallic LDHs of Ni-LDH (121.3 mV dec^−1^) and Fe-LDH (62.8 mV dec^−1^), the bi-metallic NiFe-LDH had a lower Tafel slope (60.2 mV dec^−1^), suggesting that bi-metallization facilitated a decrease in the Tafel slope. After introducing carbon nanotubes, NiFe-LDH@CNT exhibited an extraordinarily low Tafel slope of 51.36 mV dec^−1^, which is obviously better than that of NiFe-LDH (65.7 mV dec^−1^). This result demonstrates that, by combining bi-metallization and the conductive skeleton strategy, superior catalytic activity of NiFe-LDH@CNT can be obtained.

**Figure 6:**
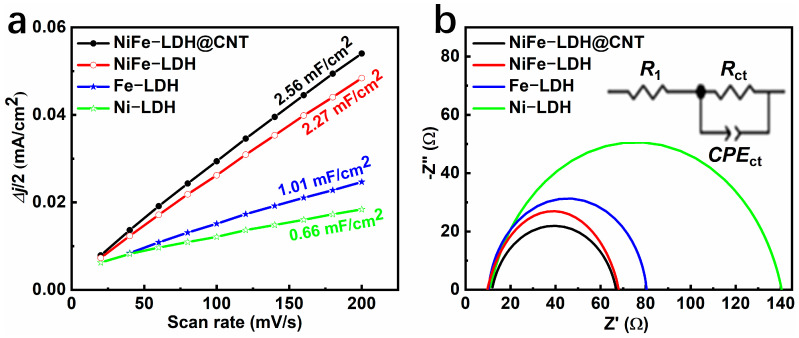
(**a**) *C*_dl_ data and (**b**) EIS spectra of Ni-LDH, Fe-LDH, NiFe-LDH, and NiFe-LDH@CNT.

To accurately depict the alterations in the material, we conducted measurements of the electrochemical active surface area (ECSA) within a potential window devoid of Faradaic processes. Generally, elevated values of double-layer capacitance (*C*_dl_) indicate an increased ECSA, which corresponds to a greater number of active sites and a more robust OER [27]. The *C*_dl_ values of Ni-LDH, Fe-LDH, NiFe-LDH, and NiFe-LDH@CNT were derived from the CV curves (Figure S1). Figure 6a illustrates that the *C*_dl_ values of Ni-LDH, Fe-LDH, NiFe-LDH, and NiFe-LDH@CNT were 0.66, 1.01, 2.27, and 2.56 mF cm^−2^, respectively. One can observe that, compared to the monometallic LDHs of Ni-LDH (0.66 mF cm^−2^) and Fe-LDH (0.66 mF cm^−2^), the bi-metallic NiFe-LDH showed a much larger *C*_dl_ value (2.27 mF cm^−2^), which suggests that bi-metallization generates many more active sites; compared to NiFe-LDH, the *C*_dl_ value of NiFe-LDH@CNT was further increased. This can be explained by the introduction of carbon nanotubes and the interconnection between NiFe-LDH and the carbon nanotubes, which further generated additional active sites. Among these samples, NiFe-LDH@CNT possessed the highest *C*_dl_ value of 2.56 mF cm^−2^, confirming that it would have the best OER performance. 

Subsequently, electrochemical impedance spectroscopy (EIS) was used to assess the reaction kinetics. As shown in Figure 5b, the EIS plot was fitted using the Z-View software (Zview2.8). The semicircle diameters of the EIS plots, which represent the values of the charge transfer resistance (*R*_ct_), showed the complexity and kinetics of the reaction. Notably, NiFe-LDH@CNT substantially displayed a lower *R*_ct_ value compared to the other samples, implying faster charge transfer rates, which is consistent with observations from the Tafel curves (as depicted in Figure 4b). Specifically, the *R*_ct_ values of Ni-LDH, Fe-LDH, NiFe-LDH, and NiFe-LDH@CNT were 129.08, 69.25, 57.24, and 55.93 Ω, respectively. It was obvious that the bi-metallic NiFe-LDH showed lower *R*_ct_ values than those of Ni-LDH or Fe-LDH. This superiority may have been due to the fact that the synergistic interactions between Ni and Fe could have resulted in a relatively loose electronic structure and low resistivity. Compared to NiFe-LDH, NiFe-LDH@CNT displayed a smaller value for *R*_ct_ (55.93 Ω), which indicates that the highly conductive, CNT-interconnected, bi-metallic LDH composite enabled faster charge transfer and better OER performance. This shows that NiFe-LDH@CNT has a good potential for practical applications. 

**Figure 7:**
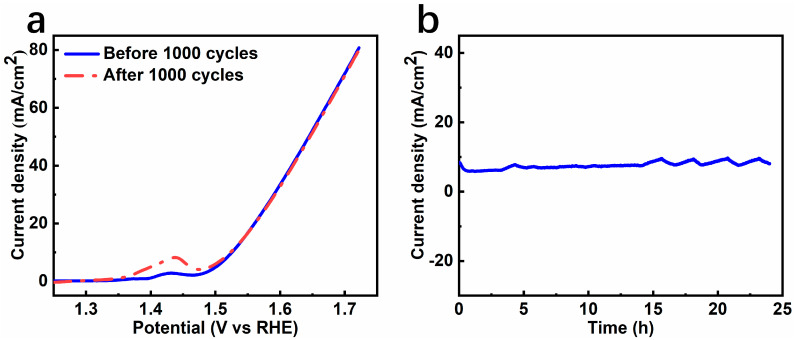
Stability of NiFe-LDH@CNT. (**a**) LSV curves before and after 1000 cycles. (**b**) *i-t* test at a fixed voltage of 0.604 V.

The stability of the electrocatalyst under harsh alkaline conditions is crucial for practical applications. With this in mind, the cycling stability and long-term durability of NiFe-LDH@CNT in a harsh alkaline electrolyte were investigated. As depicted in Figure 7a, the LSV plot obtained for NiFe-LDH@CNT after 1000 contentious CV cycles on a GC electrode displayed a nearly identical profile to the LSV plot obtained before the cycling test, indicating significant cycling stability. The plot suggests that, when the potential exceeds 1.5 V, only minimal degradation is observed, along with a slightly elevated Ni^2+^ to Ni^3+^ oxidation peak, signifying its stable electrocatalytic OER activity. Moreover, a chronoamperometric test was carried out by depositing the catalyst ink onto nickel foam. As shown in Figure 7b (*i*~*t* curve), at a fixed voltage of 0.604 V (compared to the Hg/HgO electrode) to obtain a current density of 10 mA/cm^2^, the current density retention could be up to 94.45% after 24 h, suggesting excellent long-term stability during the OER process in an alkaline solution. The impressive catalytic activity and good durability of NiFe-LDH@CNT indicate its potential for practical applications. 

## 3. Materials and Methods

### 3.1. Materials

Analytical-grade nickel(II) nitrate hexahydrate, iron(III) nitrate nonahydrate, 5 wt% CNT paste in N-methyl-2-pyrrolidone (CNTs in NMP), methanol, and ethanol were obtained from Aladdin, Co., Ltd. Shanghai, China, while the 5 wt% Nafion ink was purchased from DuPont Co., Wilmington, DE, USA. These reagents were used without additional purification. Deionized (DI) water (resistivity = 18.2 MΩ cm) and ethanol served as the washing solvents throughout the entire investigation.

### 3.2. Synthesis

All transition-metal-based, layered, double hydroxide samples in this work, including the monometallic Ni-LDH and Fe-LDH, the bi-metallic NiFe-LDH, and the composite of NiFe-LDH and carbon nanotubes (NiFe-LDH@CNT), were synthesized via a facile co-precipitation method at ambient temperature. Nickel(II) and/or iron(III) salts and dimethylimidazole (2 MI) were used as the precursors, and a mixture of methanol and water acted as the reaction medium. The detailed synthesis methods of Ni-LDH, Fe-LDH, NiFe-LDH, and NiFe-LDH@CNT are described as follows.

#### 3.2.1. Synthesis of Ni-LDH

Monometallic Ni-LDH was synthesized by a co-precipitation reaction of nickel(II) salt with 2 MI in a water–methanol solution at ambient temperature. Typically, 1168 mg of 2 MI was dissolved in 70 mL of methanol, while simultaneously, 1453.95 mg of nickel(II) salt was dissolved in the same amount of a methanol-and-DI-water mixed solution (4:1 volume ration). The two solutions were then mixed under continuous stirring for 10 min. The resulting mixture was left undisturbed for 4 h at ambient temperature. The obtained precipitate was subsequently filtered and washed two times using ethanol and water, followed by lyophilization. The obtained solid product was named Ni-LDH and sealed for further characterizations and an electrocatalytic activity test.

#### 3.2.2. Synthesis of Fe-LDH

Monometallic Fe-LDH was synthesized at room temperature via a co-precipitation reaction of iron(III) salt and 2 MI in a water–methanol solution. Following a similar process to the Ni-LDH synthesis, a methanolic solution containing 1168 mg of 2 MI was combined with a water–methanol mixture containing 1399.25 mg of iron(III) salt. The two solutions were then mixed under continuous stirring for 10 min and left undisturbed for 4 h. The resulting precipitate (denoted as Fe-LDH) was collected using the same procedure outlined earlier for Ni-LDH.

#### 3.2.3. Synthesis of NiFe-LDH

Bi-metallic NiFe-LDH (Ni:Fe molar ratio = 3:1) was also synthesized following the co-precipitation method as described above for monometallic LDHs. Precisely, the methanolic solution containing 1168 mg of 2 MI was combined with a water–methanol mixture containing 1090.46 mg of nickel(II) and 349.81 mg of iron(III) salts, and stirred at ambient temperature for a specified time (4 h). The resulting precipitate was allowed to settle down. The obtained precipitate was labelled NiFe-LDH and collected by washing with ethanol and water as described above. 

#### 3.2.4. Synthesis of NiFe-LDH@CNT

The synthesis procedure for NiFe-LDH@CNT followed a similar approach to the preparation of NiFe-LDH, with additional 5 wt% CNTs uniformly dispersed in a methanol-and-water mixture prior to the addition of nickel(II) and iron(III) salts in a 3:1 molar ratio. Typically, 1168 mg of 2 MI was dissolved in 70 mL of methanol, and at the same time, 2.8 g of CNT paste (5 wt% CNTs in NMP) was thoroughly dispersed in 70 mL of a water–methanol mixture by rigorously stirring for at least 30 min. Following this, 1090.46 mg of nickel(II) and 349.81 mg of iron(III) salts were dissolved in the CNT-dispersed mixed solution. Subsequently, the 2 MI solution was combined with the solution containing nickel(II), iron(III), and CNTs, continuously stirred for 10 min, and left undisturbed for 4 h at ambient temperature. The ensuing precipitate was filtered, thoroughly washed multiple times with ethanol and DI water, and subjected to drying using a freeze dryer.

### 3.3. Structural Characterizations 

The in-depth examination of the structural properties involved studying the X-ray diffraction (XRD) patterns for NiFe-LDH@CNT, NiFe-LDH, and CNTs. This analysis utilized a Bruker D8 Advance powder X-ray diffractometer, operating at 40.0 kV and 40.0 mA (radiation source = Co Kα and wavelength = 1.79026 Å), from a scan range of 10° to 80° (scan rate = 4°/min). To obtain the Raman spectrum of the NiFe-LDH@CNT sample, a confocal laser Raman microscope was employed (Renishaw Corporation; excitation wavelength = 532 nm). Observing the morphology of the materials was achieved through SEM imaging using a Hitachi SU8020 microscope (Hitachi Ltd., Tokyo, Japan). Furthermore, the microstructure of the materials was thoroughly investigated by utilizing TEM, HR-TEM, and TEM-EDS. These techniques were executed using FEI Tecnai G2 F30 (FEI Ltd., Hillsboro, USA) and XPLORE (Oxford Instruments, Abingdon, UK). The acceleration voltages for the TEM imaging and TEM-EDS mapping were set to 300 kV. An X-ray photoelectron spectroscopy (XPS) analysis was conducted using an ESCALAB 250XI instrument (Thermo Fisher Scientific Inc., Waltham, MA, USA). The excitation source utilized was Al Kα radiation, generating photons with an energy of 1486.6 eV. The binding energies were adjusted and aligned relative to the C 1s peak, which was established as the baseline, showcasing the lowest binding energy at 284 eV. 

### 3.4. Electrochemical Characterizations 

Detailed electrochemical analyses were carried out using a CHI-660D electrochemical workstation (CH Instruments Co., Ltd., Shanghai, China). The experimental setup comprised a reference electrode (Hg/HgO electrode, CHI152), a counter electrode (graphite rod), and a working electrode (glassy carbon electrode or GCE) loaded with the catalyst ink.

The catalyst ink was prepared by mixing 750 µL of DI water, 250 µL of ethanol, 50 µL of Nafion ink, and 4 mg of different catalyst samples, followed by ultrasonication for 30 min. Subsequently, the well-dispersed catalyst ink (5 µL) was dropped onto the center of the pre-treated GCE (polished with Al_2_O_3_) and dried at 60 °C until a layer of the catalyst film attached to the center. After cooling down to ambient temperature, the electrochemical tests were initiated. 

The potential measurements were adjusted to the potential versus a reversible hydrogen electrode (RHE) using the conversion equation: E vs. RHE = E vs. Hg/HgO + 0.0592 × pH + 0.098. The pH of the 1.0 M KOH electrolyte was determined to be 13.96. For the linear sweep voltammetry (LSV) tests, scans were performed within the range of 0.923 to 1.722 V versus RHE (scan rate = 5 mV/s), following 20 cycles of cyclic voltammetry (scan rate = 0.1 V/s). Electrochemical impedance spectroscopy (EIS) was conducted at a potential corresponding to a current density of 10 mA/cm^2^, covering a frequency range from 1.0 × 10^−3^ to 1.0 × 10^−5^ Hz. The electrochemically active surface-area tests were based on the double-layer capacitance theory using the cyclic voltammetry (CV) method. The measurements were executed within the voltage range of 0.35 to 0.45 V versus RHE, employing scan rates ranging from 20 to 200 mV/s. The stability tests consisted of two parts: an *i*~*t* test recorded at a current density of 10 mA/cm^2^ and cyclic voltammetry measurements involving 1000 cycles of CV. Foam nickel (NF, 1 cm × 1 cm) was used as the load material in these tests. Since the electrolyte concentration was high (1 M KOH), no IR compensation was considered in any of the polarization curves.

## 4. Conclusions

In order to effectively boost the OER performance of transition-metal-based layered double hydroxides, we present a synergistic approach by combining crystalline structure control via the synthesis conditions and the OER performance enhancement strategy of electronic structure modification via bi-metallization and the construction of a conductive skeleton. A facile, one-pot co-precipitation approach was successfully developed to swiftly synthesize amorphous NiFe-layered double hydroxides interconnected with carbon nanotubes (NiFe-LDH@CNT) at room temperature. This room-temperature synthesis not only offers a cost-effective and environmentally sustainable approach, but also allows for the advantageous amorphous structure of NiFe-LDH without distinct crystalline boundaries. This structural characteristic significantly contributes to the long-term stability observed during the OER. The bi-metallic composition of NiFe-LDH plays a vital role in modifying the electronic configuration and creating additional catalytic sites. Furthermore, the integration of carbon nanotubes (CNTs) aids in forming a nanoporous structure, enabling efficient ion diffusion and oxygen release. The highly conductive network of carbon nanotubes provides effective conductive paths and establishes a well-structured interface with layered double hydroxides, facilitating electron transfer. The combined advantages of these structural and compositional aspects culminate in outstanding OER activity for NiFe-LDH@CNT, exemplified by a minimal onset overpotential of 255 mV, an impressively low Tafel slope of 51.36 mV dec^−1^, and significant long-time operability. In summary, this work not only introduces a strategy for designing a well-considered nanoarchitecture, but also manifests a simple synthesis route to achieve non-precious metal-based amorphous structures for OER catalysis. This approach holds great promise and presents economically viable, highly efficient, and stable OER catalysts for practical applications.

## Data Availability

The data are available from the corresponding authors upon request.

## Supplementary Materials

The following supporting information can be downloaded at: https://www.mdpi.com/article/10.3390/molecules28217289/s1. Figure S1: Voltammograms of Ni-LDH, Fe-LDH, NiFe-LDH, and NiFe-LDH@CNT; Table S1: The fitted peak data of XPS of NiFe-LDH@CNT.
