# Supplementary material for: Room-Temperature Synthesis of Carbon-Nanotube-Interconnected Amorphous NiFe-Layered Double Hydroxides for Boosting Oxygen Evolution Reaction
Source: Molecules. 2023 Oct 27;28(21):7289. doi: 10.3390/molecules28217289 (PMC10648594; doi:10.3390/molecules28217289)
Supplement: Supplementary file 1 [file molecules-28-07289-s001.zip › molecules-2672662-supplementary.pdf]

Supporting Information

Room-Temperature Synthesis of Carbon-Nanotube-Interconnected Amorphous NiFe-Layered Double Hydroxides for Boosting Oxygen Evolution Reaction

Zhuo Chen [1,†], Qiang Qu [1,†], Xinsheng Li [2], Katam Srinivas [2], Yuanfu Chen [2,*] and Mingqiang Zhu [1,*]

[1] College of Mechanical and Electronic Engineering, Northwest A&F University, Yangling 712100, China
[2] State Key Laboratory of Electronic Thin Films and Integrated Devices, School of Integrated Circuit Science and Engineering, University of Electronic Science and Technology of China, Chengdu 610054, China
* Correspondence: yfchen@uestc.edu.cn (Y.C.); zmqsx@nwsuaf.edu.cn (M.Z.)
† These authors contributed equally to this work.

XPS fitting data

The fitted peak data of XPS of NiFe-LDH@CNT are summarized in Table S1.

Table S1. The fitted peak data of XPS of NiFe-LDH@CNT

Elements	Peak (BE) eV	Peak (BE) eV	Peak (BE) eV	Peak (BE) eV	Peak (BE) eV	Peak (BE) eV	Peak (BE) eV
Ni 2p	854.99	856.40	860.80	863.46	872.52	874.68	879.03
Fe 2p	704.67	711.46	717.25	724.63	731.88		
C 1s	284.00	285.17	287.29				

CV curves for calculating C_{dl}

The C_{dl} data can be calculated from the CV curves. The CV curves of Ni-LDH, Fe-LDH, NiFe-LDH, and NiFe-LDH@CNT are shown in Figure S1.

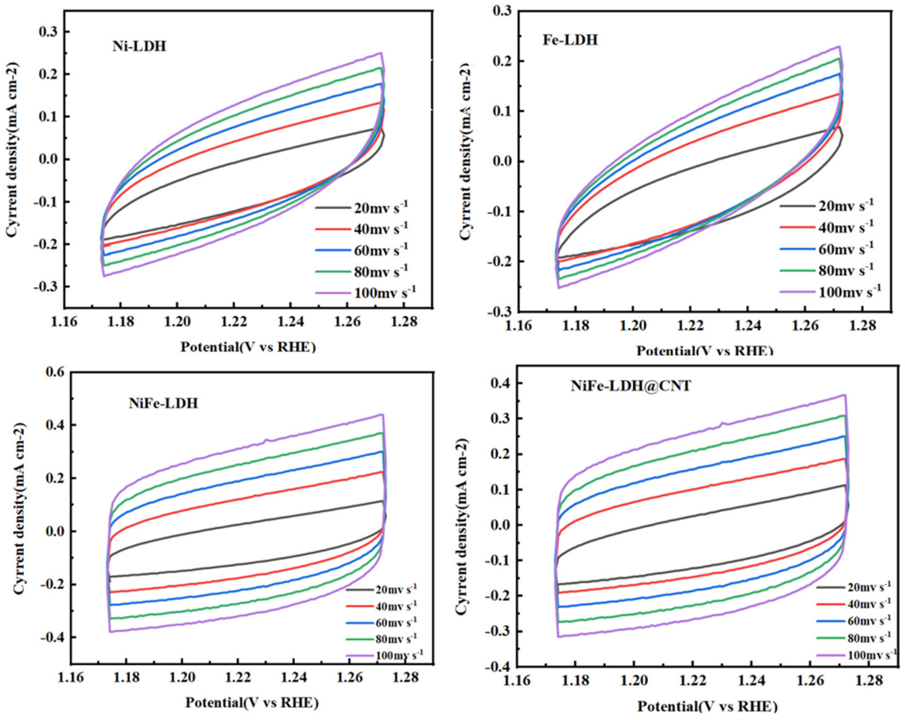

Figure S1. Voltammograms of Ni-LDH, Fe-LDH, NiFe-LDH, and NiFe-LDH@CNT for OER in alkaline electrolyte.
